# Artificial intelligence-based detection of epimacular membrane from color fundus photographs

**DOI:** 10.1038/s41598-021-98510-x

**Published:** 2021-09-29

**Authors:** Enhua Shao, Congxin Liu, Lei Wang, Dan Song, Libin Guo, Xuan Yao, Jianhao Xiong, Bin Wang, Yuntao Hu

**Affiliations:** 1 Department of Ophthalmology, Beijing Tisnghua Changgung Hospital, School of Clinical Medicine, Tsinghua University, Beijing, China; 2 Beijing Eaglevision Technology Co., Ltd, Beijing, China

**Keywords:** Retinal diseases, Software

## Abstract

Epiretinal membrane (ERM) is a common ophthalmological disorder of high prevalence. Its symptoms include metamorphopsia, blurred vision, and decreased visual acuity. Early diagnosis and timely treatment of ERM is crucial to preventing vision loss. Although optical coherence tomography (OCT) is regarded as a de facto standard for ERM diagnosis due to its intuitiveness and high sensitivity, ophthalmoscopic examination or fundus photographs still have the advantages of price and accessibility. Artificial intelligence (AI) has been widely applied in the health care industry for its robust and significant performance in detecting various diseases. In this study, we validated the use of a previously trained deep neural network based-AI model in ERM detection based on color fundus photographs. An independent test set of fundus photographs was labeled by a group of ophthalmologists according to their corresponding OCT images as the gold standard. Then the test set was interpreted by other ophthalmologists and AI model without knowing their OCT results. Compared with manual diagnosis based on fundus photographs alone, the AI model had comparable accuracy (AI model 77.08% vs. integrated manual diagnosis 75.69%, χ^2^ = 0.038, *P* = 0.845, McNemar’s test), higher sensitivity (75.90% vs. 63.86%, χ^2^ = 4.500, *P* = 0.034, McNemar’s test), under the cost of lower but reasonable specificity (78.69% vs. 91.80%, χ^2^ = 6.125, *P* = 0.013, McNemar’s test). Thus our AI model can serve as a possible alternative for manual diagnosis in ERM screening.

## Introduction

Epiretinal membranes (ERMs) are the abnormal formation of non-vascular cellular or fibrous membrane at the inner layer of retina^1^. Typically, when the membrane locates around macular region, it is called epimacular membranes or premacular fibroplasis. Most ERMs are asymptomatic and remain stable or even regress automatically in absence of medical intervention^2^. However, there are cases that develop rapidly within months, causing blind spot or scotoma^3^. Further contraction of the membrane may tear the retina and lead to severe outcomes such as retinal hemorrhage, retinal edema, or even macular holes^4,5^. Reported prevalence of ERM ranges from 3.4% to 39% in different populations^6–15^. These published incidences can be under-estimated, due to difficulty in diagnosis of secondary ERM with the presence of primary ocular diseases.

Clinical management of ERM was observed at early asymptomatic stage or surgical intervention at late stage^1^. Surgical removal of ERM can restore retinal structure and improve visual acuity, but there is potentially residual impairment of visual function^16^. Better visual acuity improvements have been found with: (i) shorter latency between symptom onset and surgery^17^; (ii) better preoperative visual acuity^17–19^; (iii) less central foveal thickness measured by OCT^20,21^; (iv) maintained integrity of retinal structure^18–20,22,23^; (v) thinner ganglion cell inner plexiform layer^24^; (vi) absence of co-existing maculopathy^25^. Therefore, early detection of ERM and timely surgical intervention are crucial to achieving better visual outcomes.

With the high prevalence and possible severe consequence of the disease, fast and inexpensive approaches for ERM screening in large population are in need. Traditionally, ERMs were diagnosed and classified based on subjective symptoms and ophthalmoscopic examination^26^. Fundus photographs provide the possibility of review and compare ERM at different time points. Experienced ophthalmologists are required for correct interpretation of retinal images. Non-invasive modern examination, such as optical coherence tomography (OCT), provides high-resolution structural information of live retina and has been widely used as an essential diagnostic tool in multiple maculopathies. Cross-sectional macula-centered OCT images provide clear and intuitive structural relationship of epimacular membrane and the neuroretina, and have been proved to be more sensitive than other clinical examinations in ERM diagnosis^27^. Furthermore, linear scanning across the macular fovea provides multiple quantifiable structural parameters including the central foveal thickness, the volume of macular edema, and the ERM thickness^18,21,28,29^. Based on these quantifiable data, several OCT-based ERM classification systems have been established, contributing to a better understanding of ERM. However, the promotion of OCT in primary hospitals is difficult due to price issues.

Deep learning-based artificial intelligence (AI) has been widely applied in assisting detection and staging of common ocular disorders with distinctive features, such as diabetic retinopathy^30–33^, glaucoma^34^, cataract^35^, age-related macular degeneration^36,37^, and retinopathy of prematurity^38–40^. Unlike the traditional coding process where methodology has already been established by programmers before coding, decision-making strategy is figured out by deep-learning algorithms throughout the coding process, which means deep learning algorithms may detect novel features that highly related to disease diagnosis or staging^41^. AI models have shown reliable performance in detection of ERM based on OCT images^42,43^. However, currently there is no validated AI model for ERM detection based on fundus photographs, which is of easier access and lower cost. In this study, we compared manual or AI detection of ERM based on fundus photographs with the standard diagnosis of ERM based on OCT and other clinical information, validating the use of AI model in assisting detection of ERM using fundus photographs.

## Methods

### Case recruitment

**Figure 1:**
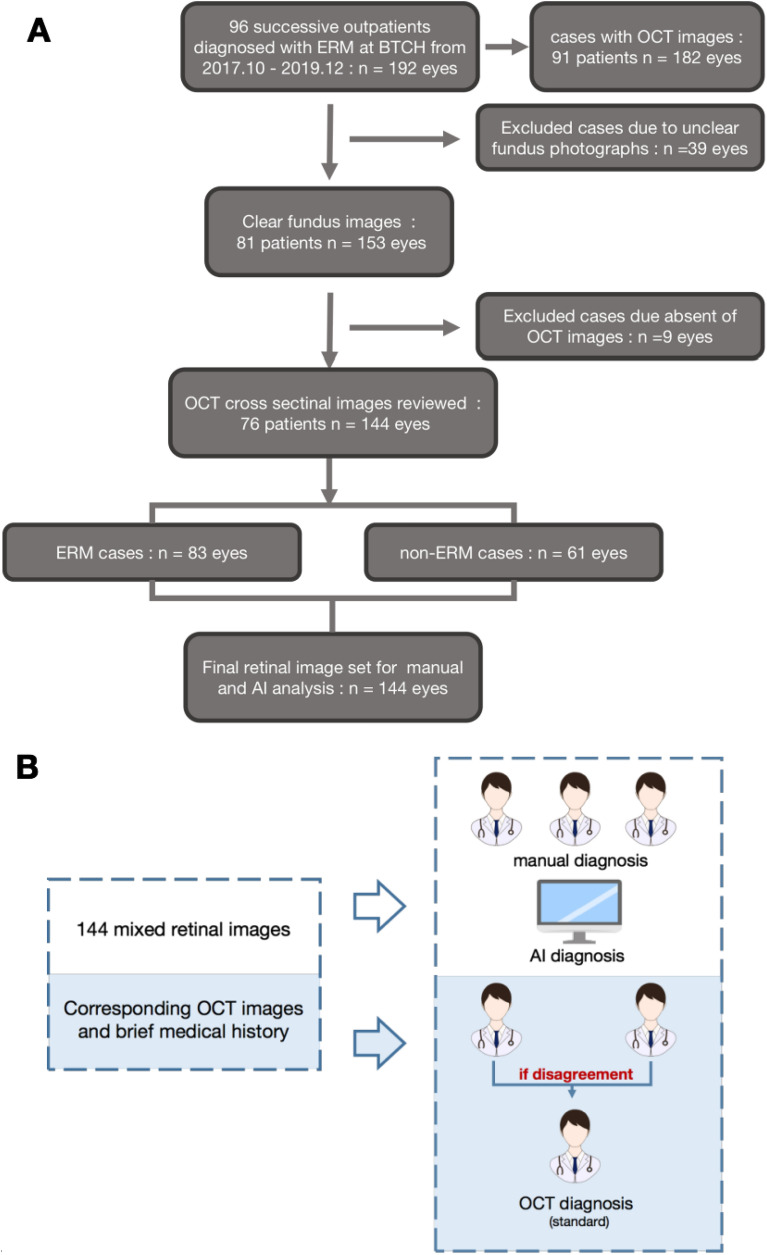
Experimental design. (**A**) Inclusion and exclusion of ERM case and non-ERM sex and age matched group are demonstrated in the flow chart. (**B**) Simultaneously, mixed retinal image set were evaluated by 3 ophthalmologists and AI model respectively. Result from manual diagnosis and AI model were compared with standard OCT diagnosis. *ERM* epiretinal membrane, *BTCH* Beijing Tsinghua Changguang Hospital.

Total 96 successive outpatients diagnosed with ERM from 2017 October to 2019 December at Beijing Tsinghua Changgung Hospital were recruited in this retrospective study (Fig. 1). Their basic information, brief medical history, fundus photographs of both eye (192 eyes) (macular-centered, by Canon CR-2 Digital Retinal Camera (Canon, Long Island, NY, US)), and ipsilateral OCT images (vertical and horizontal cross section at macular, by Heidelberg Engineering OCT Spectralis (Heidelberg Engineering, Heidelberg, Germany)) were anonymized and acquired from the hospital information system. This study was approved by the Ethical Committee of Beijing Tsinghua Changgung Hospital. The Ethical Committee of Beijing Tsinghua Changgung Hospital waived the need for informed consent from the participants. All methods were carried out in accordance with the Declaration of Helsinki and the International Ethical Guidelines for Biomedical Research Involving Human Subjects.

### ERM diagnosis by OCT images

**Figure 2:**
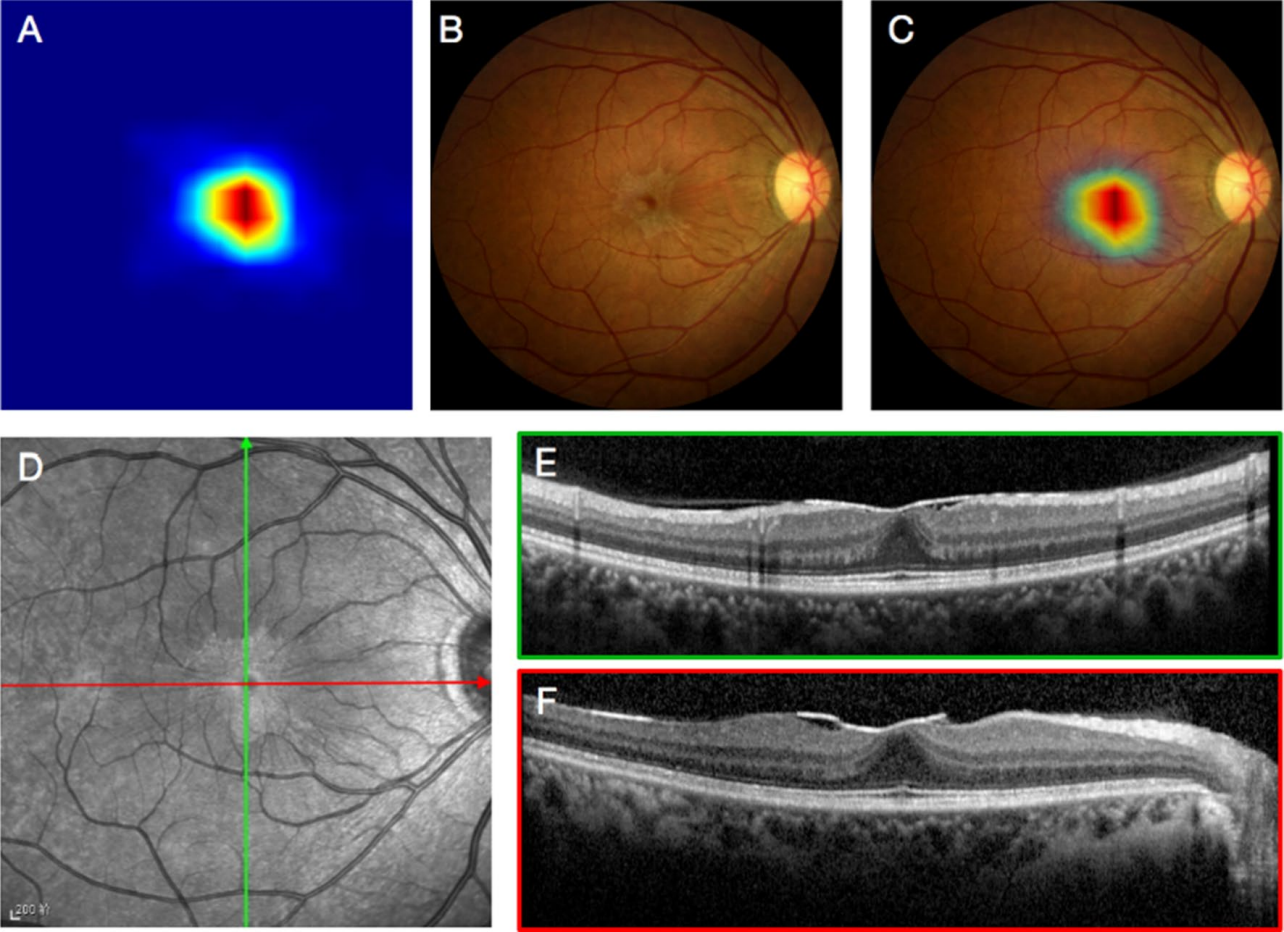
An example of accurate recognition of ERM in fundus photograph by AI model. (**A**) Grad-CAM image showing suspected location of ERM. (**B**) Original fundus photograph. (**C**) Merged image of fundus photograph and Grad-CAM. (**D**) Infrared fundus image of the same ERM eye, with red and green lines representing location of cross sectional OCT images. (**E**, **F**) Cross sectional OCT images across fovea showed ERM.

Corresponding OCT images of all recruited retinal images (including both ERM and non-ERM groups) were examined individually by two experienced ophthalmologists. Sex, age, and brief medical history of the patients were provided to the examiners in order to improve diagnostic accuracy. Both examiners gave binary diagnosis of ERM or non-ERM. When the two examiners reported contradicting diagnoses, a third specialist re-examined the corresponding OCT images and made the final OCT diagnosis. Figure 2D–E showed typical OCT images of ERM.

### Manual and AI interpretation of fundus photographs

Fundus photographs of ERM and non-ERM eyes were mixed together and randomly labeled with successive numbers. Resulted retinal image set was interpreted independently by three different ophthalmologists without knowing the sex, age, medical history, and OCT result of the cases. The three ophthalmologists consist of one resident doctor, one attending, and one retina specialist, representing different experience levels in ERM diagnosis and management. Mixed fundus photographs set were analyzed by AI model simultaneously. Figure 2A–C were examples of visualized ERM detection of AI model in form of Grad-CAM^44^. The AI model outputs binary result (ERM or non-ERM).

### Brief introduction of the AI model

The AI model used in our ERM recognition is a multiply retinal diseases classification model based on a deep neural network (DNN). Neural network inspired by animal and human visual system mimics how the human brain works in processing data and pattern abstraction. Due to its depth and massive layers, DNN has huge representation power to learn visual features of retinal diseases and discriminate them effectively. The disease classification model adapted a novel backbone neural network that combines Inception-Resnet-v2^45^ and Xception^46^ architecture. An n-way binary classification layer is added to the top of the backbone network, which yields the probability of each disease. Tensorflow machine learning framework is used in the training model^47^. The AI model used in this study was previously trained with a different dataset consisting of 207,228 fundus photographs collected from 16 clinical settings with different disease distributions across China, with an average age of 40.83 ± 15.43 (registered with ClinicalTrials.gov, NCT04213430)^48^. It was annotated by an expert ophthalmologist team as 14 retinal abnormalities including ERM. Data augmentation methods during the training included Inception network family-related data sampling^49^ and random rotation that imitates intrinsic similarity of fundus image. Before image analysis, any given RGB fundus photograph is cropped and resized into 585 × 585 square with only the region around the posterior pole reserved. After analysis, the model returns a 1 × n vector whose every element represents the probability of a certain disease (ranging from 0 to 1). For each disease, a positive result is assigned to the image if the corresponding element exceeds the threshold of the disease. In this study, only the element linked to ERM was taken into consideration.

### Statistical analysis

Performance of both manual and AI interpretations were calculated with respect to standard diagnosis based on OCT images. The accuracy of manual and AI detection for ERM were calculated using the equation accuracy = (TP + TN)/ALL (TP: true positive; TN: true negative; ALL: total sample size). Sensitivity and specificity of manual and AI detection were also calculated, using the equation sensitivity = TP/(TP + FN), specificity = TN/(TN + FP) (TP: true positive, TN: true negative, FP: false positive, FN: false negative). Kappa (κ) statistics were used to quantify the degree of agreement comparing different individual manual analyses, summarized manual results, AI interpretation with the standard OCT result. Statistical significance was considered at *P* < 0.05.

## Results

### Basic information of recruited cases

A total of 96 patients has been recruited in this study. Among them, 48 (50%) are female and 48 (50%) are male, with an average age of 66.75 ± 10.03 and 72.25 ± 12.09 respectively (Table 1). Our reported even distribution of ERM patients in different sex groups is in consensus with the previously reported consistent prevalence in both male and female groups. Among all these outpatients, 69 (71.87%) of them had symptoms of blurred vision, decreased visual acuity, or metamorphopsia. 27 (28.13%) of them had no obvious symptoms and the ERM was found accidentally through physical examination or during following up of other ocular diseases. The most frequent comorbidity in our recruited group was age-related cataract. Other comorbidities include macular edema, diabetic retinopathy, retinal detachment, and macular hole.

**Table 1:** Basic information of recruited outpatients.

	Male	Female	Total	*P* value*	95% CI
Number of patients	48 (50%)	48 (50%)	96	–	–
Average age	66.75 ± 10.03	72.25 ± 12.09	69.2	0.015	− 8.029 to − 2.971

*Independent t-test was employed between genders.

### OCT diagnosis results

**Figure 3:**
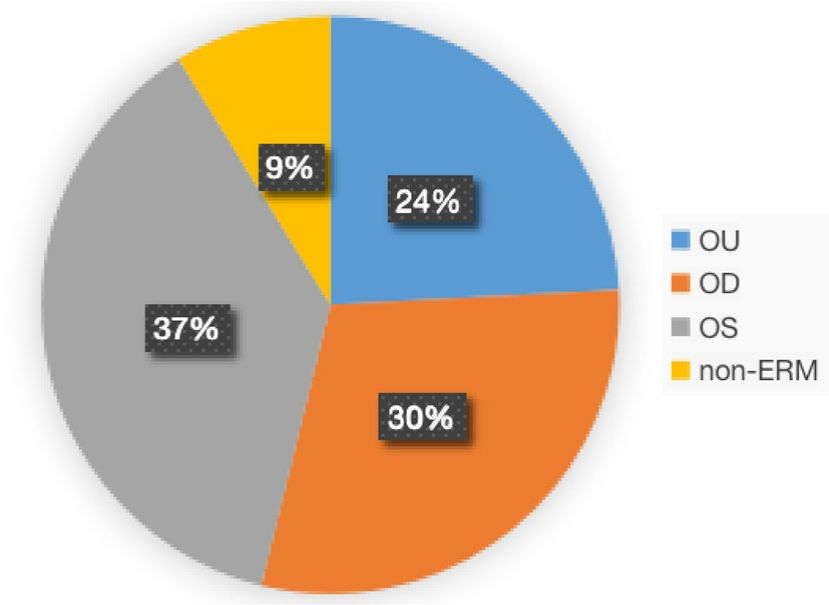
Rate of affected eyes in recruited population. The above pie chart illustrated composition of recruited population in terms of different affected eyes diagnosed by OCT. Among 91 patients with their OCT images re-examined by ophthalmologists, 22 patients were diagnosed with ERM in both eyes; 27 were diagnosed with right-eye ERM only; 34 were diagnosed with left-eye ERM only; 8 were confirmed as non-ERM. Percentages of each kind were labeled next to its corresponding sector on the chart.

Totally 96 outpatients (192 eyes) were recruited in the beginning, among which 91 patients (182 eyes) have done OCT and were included in further analysis (Fig. 1A). All acquired OCT images were re-examined by retinal specialists in order to obtain standard ERM diagnosis. For all the OCT-examined patients, 24% of them have both eyes diagnosed with ERM. Left-eye only and right-eye only ERM took up 30% and 37% of the population respectively (Fig. 3).

After elimination of unclear fundus photographs due to cataracts or vitreous opacity, 153 fundus images from 91 patients were reserved. Among them, 144 eyes have corresponding OCT images and their diagnosis of ERM was re-confirmed by experienced ophthalmologists based on OCT images as well as basic information and brief medical history of the patients (Fig. 1B). Eventually, 83 affected eyes and 61 non-ERM eyes were figured out from the image set. There is no significant difference with right or left eye between the ERM and non-ERM groups (chi-square test, χ^2^ = 0.006, *P* = 0.9383) (Table 2). The mixture of both ERM and non-ERM eyes was reserved for subsequent analysis.

**Table 2:** OCT diagnosis result.

	OD*	OS*	Total	χ^2^	*P* value
ERM	43	40	83	0.006	0.9383
Non-ERM	32	29	61		
Total	75	69	144		

*OD right eye, *OS* left eye.

### Manual and AI interpretation of fundus photograph set

Three ophthalmologists analyzed the fundus photograph set individually. The diagnostic accuracy of the three examiners were 74.31 ± 1.82%, 73.61 ± 1.84%, and 72.22 ± 1.87% (95% CI) respectively. Kappa tests between any two of them showed intermediate to high consistency among the three individual analyses. (ophthalmologist 1 & 2: kappa = 0.631, z value = 7.909, *P* = 0.000****; ophthalmologist 1 & 3: kappa = 0.529, z value = 7.081, *P* = 0.000****; ophthalmologist 2 & 3: kappa = 0.669, z value = 8.157, *P* = 0.000****). The AI model showed higher accuracy than all three manual interpretations, with an accuracy of 77.08 ± 1.75% (95% CI). However, the accuracy between any ophthalmologists and the AI model was not significantly different. (ophthalmologist 1 vs. AI: u = 0.5495, *P* > 0.05; ophthalmologist 2 vs. AI: u = 0.6836, *P* > 0.05; ophthalmologist 3 vs. AI: u = 0.9482, *P* > 0.05; u test). The highest sensitivity was achieved by the AI model (75.90%), whereas the highest specificity was found in manual interpretation (98.36%). Generally, diagnosis of ERM based on fundus photographs is an approach with high specificity (73.77–98.36%) but relatively low sensitivity (53.01–75.90%).

In order to investigate whether re-examination of the same fundus photograph would increase diagnostic accuracy, opinions from all three examiners were integrated. Diagnosis with most supporters was defined as the integrated manual diagnosis, representing the general performance of manual diagnosis. The integrated manual diagnosis resulted in an accuracy of 75.69%, which is slightly higher than the simple average of the manual accuracy (73.38%) and is higher than all of the examiners’ accuracy (Table 3). Although it is still lower than the accuracy of the AI model, no statistically significant difference was found between the accuracy of integrated manual diagnosis and the AI model. (u test, u = 0.2774, *P* > 0.05).

**Table 3:** Detailed result of different approaches.

	Ophthalmologist 1	Ophthalmologist 2	Ophthalmologist 3	Integrated manual diagnosis	AI model
ERM	78	57	45	58	76
Non-ERM	66	87	99	86	68
TP^※^	62	51	44	53	63
TN^※^	45	55	60	56	48
FP^※^	16	6	1	5	13
FN^※^	21	32	39	30	20
Accuracy	74.31%	73.61%	72.22%	75.69%	77.08%
Sensitivity	74.70%	61.45%	53.01%	63.86%	75.90%*
Specificity	73.77%	90.16%	98.36%	91.80%	78.69%**

^※^*TP* true positive, *TN* true negative, *FP* false positive, *FN* false negative. Compared with integrated manual diagnosis: *P < 0.05; **P < 0.01; McNemar’s test.

**Figure 4:**
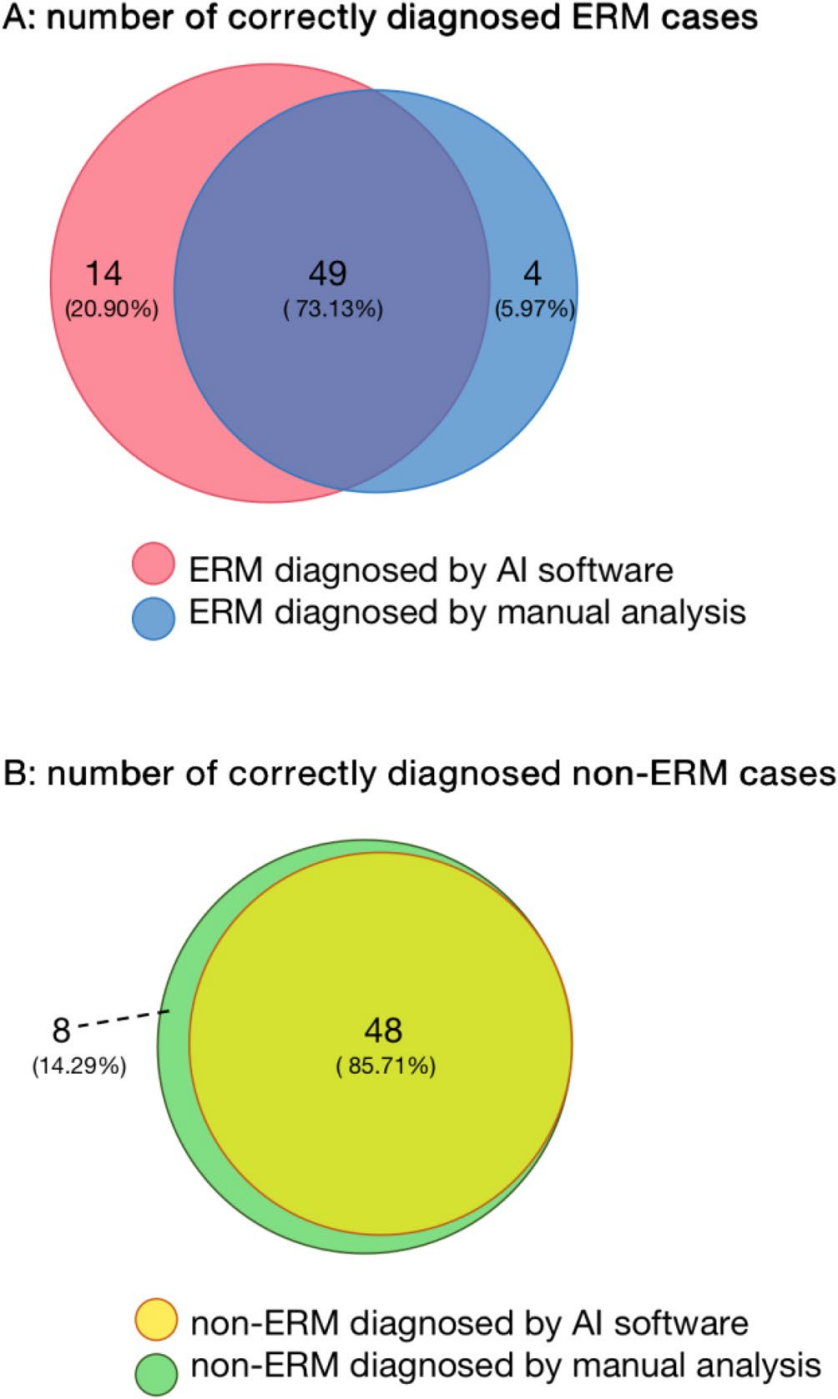
Correctly categorized ERM and non ERM by manual and AI analysis.

Further comparison between correct detection of ERM made by integrated manual analysis and AI model was shown in Fig. 4. Among all the correctly diagnosed ERM cases, 49 (73.13%) images were correctly detected by both manual approaches and AI model. 4 (5.97%) retinal images were diagnosed by manual approaches only, and another 14 (20.90%) ERM positive fundus photographs were detected by AI model only. The total number of the correctly categorized pictures will add up to 67 on either positive result. Therefore, taking both AI outputs and “manual intelligence” into consideration will obviously increase the sensitivity of diagnostic approaches. Comparison of correct elimination of ERM between integrated manual approaches and AI model were also conducted. Among all the correctly classified as non-ERM cases, 48 (85.71%) photographs were correctly eliminated both manually and with the AI model. 8 (14.29%) fundus images without ERM were detected by manual analysis only. All non-ERM cases recognized by the AI model were successfully figured out by manual diagnosis, which indicates better performance of ophthalmologists in negative prediction comparing with the AI model. Despite the differences between the two approaches, the AI model detection and manual diagnosis still greatly overlapped with each other (73.13% in correct ERM detection and 85.71% in non-ERM), which further proves the consistency between them.

**Figure 5:**
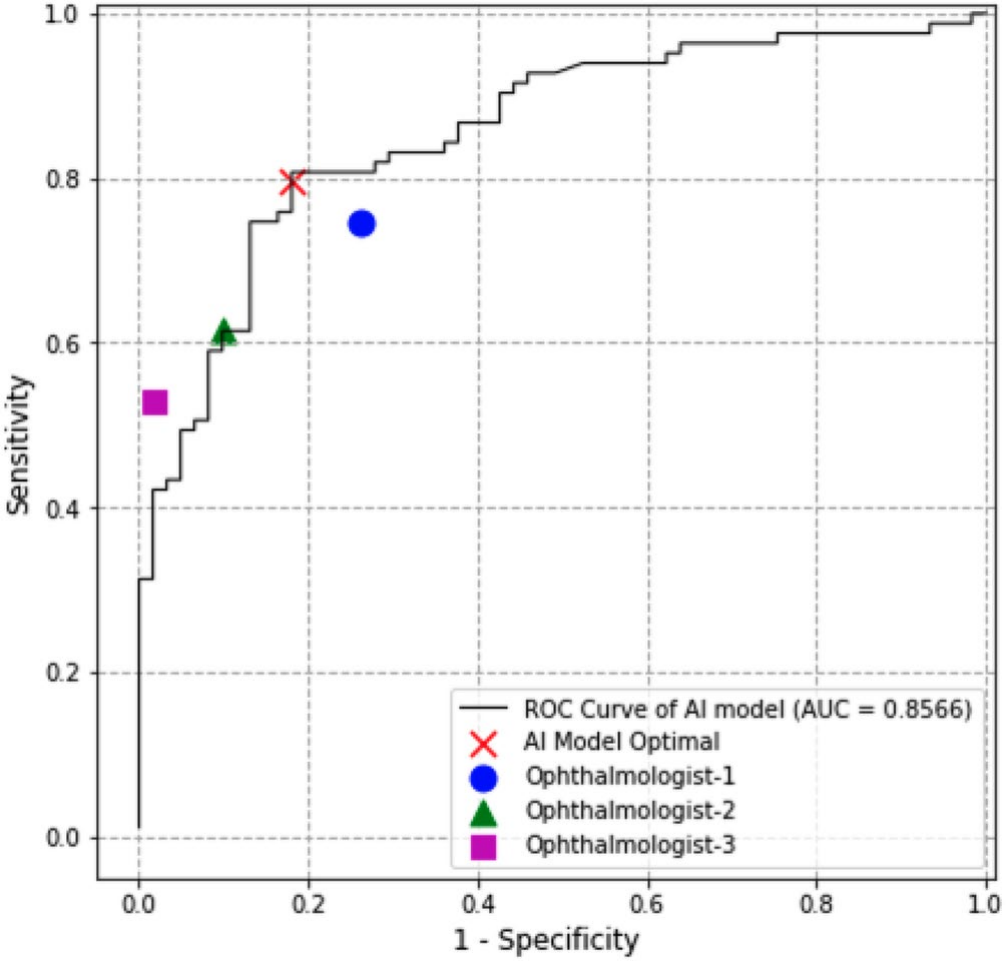
Receiver operating characteristic curve using OCT diagnosis as standard diagnosis. Operation points of manual diagnosis were illustrated on the graph, in order to compare the performance between ophthalmologists and AI model. *AUC* areas under the curve.

To eliminate the bias caused by imbalanced case numbers between ERM and non-ERM, the performance of ERM detection of the AI model was presented as receiver operating characteristic (ROC) curve and areas under the curve (AUC) (Fig. 5). The performance of AI model was comparable with ophthalmologists, with one operating point above, one under, and one on the ROC curve of AI model. AUC of the AI model was 0.8566 ± 0.061 (95% CI) using OCT diagnosis as the standard diagnosis.

### Evaluation of AI model in simulative clinical situation

**Figure 6:**
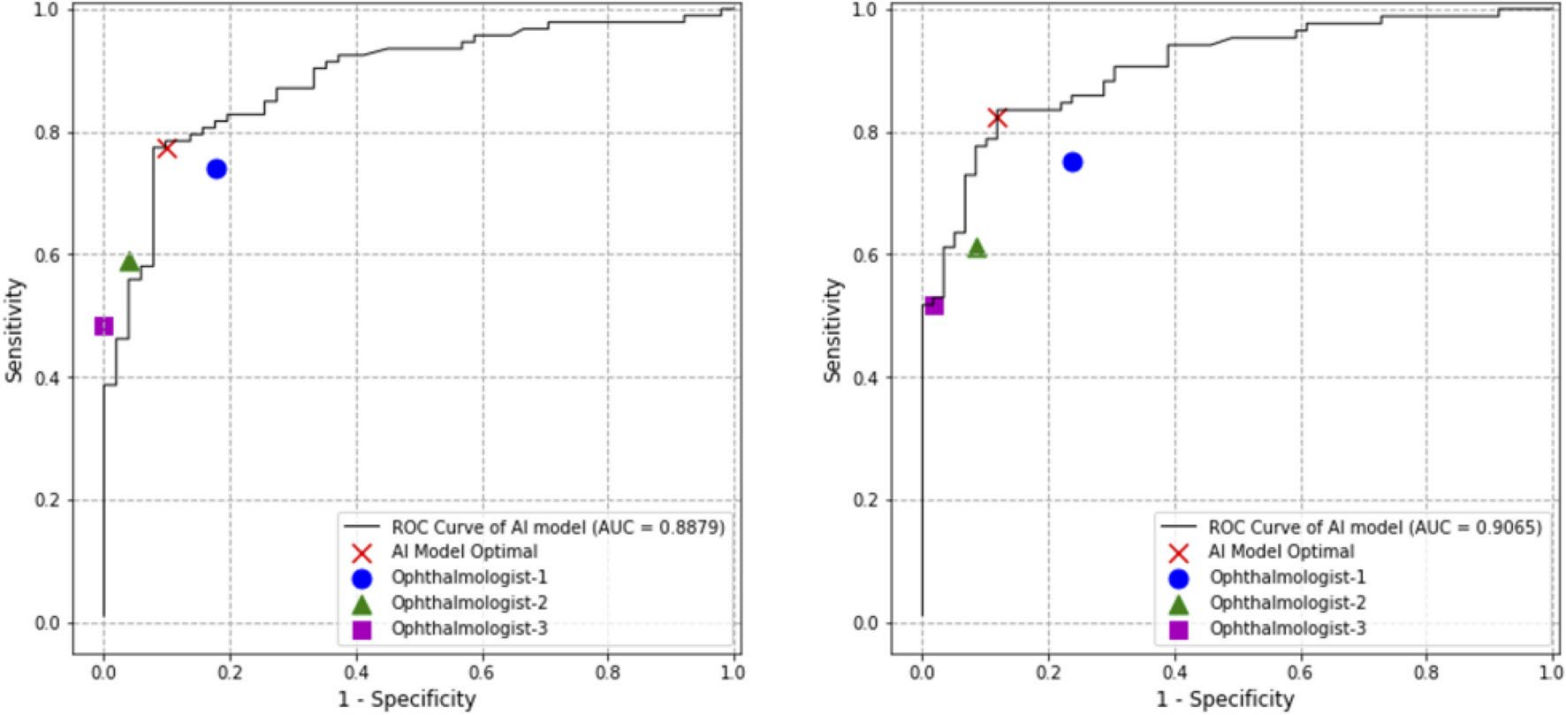
Receiver operating characteristic curve using comprehensive diagnosis as standard diagnosis. Operation points of manual diagnosis were illustrated on the graph, in order to compare the performance between ophthalmologists and AI model. *AUC* areas under the curve.

In order to mimic the diagnosis of ERM in clinics, we next provided both fundus photographs and OCT results with other clinical information to 2 retinal specialists respectively, resulted in a comprehensive diagnosis. Diagnosis made by 2 specialists showed high consistency to each other (kappa = 0.795, z value = 9.603, *P* = 0.000****). ROC curves of the AI model using either specialists’ diagnosis as standard diagnosis was illustrated in Fig. 6. Comparable performance between AI and ophthalmologists was found in both situations, with AUC of 0.8879 ± 0.0542 (specialist 1, 95% CI) and 0.9065 ± 0.0473 (specialist 2, 95% CI).

## Discussion

ERM is a retinal disease that can be commonly seen in ophthalmology clinics. Its diagnosis is mostly based on clinical findings. Variety of examinations have also been applied in ERM diagnosis: fluorescein angiography provides unique information for ERM diagnosis but is invasive and of more side effects; OCT, as a novel non-invasive imaging technique, provides an intuitive diagnosis of ERM. However, these examinations are less conducted in clinic than fundus photographs. Little studies have been reported comparing ERM diagnosis based on fundus photographs and OCT images. The only one we found in the literature reported a concordance rate of 89.13% between diagnosis based on non-mydriatic fundus images and OCT images in a group of 32 outpatients (46 eyes) with suspected idiopathic ERM^50^. Here we reported accuracy of ERM diagnosis based on fundus photographs between 72.22 and 74.31% (comparing with diagnosis based on OCT images), which is lower than the previously reported concordance rate. This may due to our inclusion of secondary ERM in the study, resulting in complicated fundus conditions. With a sensitivity of 63.86% by ophthalmologists, diagnosis of ERM based solely on fundus photographs take a huge risk of missed diagnosis. To our best knowledge, most prevalence and risk factor studies of ERM were based on diagnosis using fundus photographs^13^. The two studies that used OCT-based diagnosis were in urban and rural Chinese populations^14,51^, thus reported prevalence of ERM may be greatly underestimated in other populations.

Previously when manual labeling and AI testing were based on the same materials with manual diagnosis itself was set as standard diagnosis, it is impossible to prove AI is better than manual diagnosis since both sensitivity and specificity cannot exceed 1. Our study used a labeling method (diagnosis based on OCT images) with higher accuracy than the testing method (diagnosis based on retinal images). This kind of up-level labeling makes it possible to draw the conclusion that AI model have better performance than manual analysis and validate substitution of physicians with AI model in some conditions. Our reported AI model was based solely on retinal images. We expect higher sensitivity and specificity in ERM detection when further information, such as symptoms and medical history, are also included in AI model to improve its discrimination ability.

The application of our AI model in ERM detection, however, is not without limitation. ERM is an age-related disease. In other words, the prevalence of the ERM is higher in elder population. Prevalence of cataracts or opaque vitreous is also increased in this population, making it difficult to acquire clear retinal images. In our study, when unclear fundus images were eliminated, we reported an accuracy of 77.08% for AI detection. Most of the fundus images misclassified by AI model were of large area of darkness around macula. (Supplementary Figure 1). In the scenario of disease screening in population, however, when most people are healthy and have clear cornea, lens, and vitreous, it is easier to acquire fundus images of high quality and better performance of the AI model is expected. Moreover, the distribution of the disease and non-disease group of test image set in this study was slightly different from the dataset used in the AI model development. The average age of patients in this study is older, and the spectrum of the disease likely shifts to severe ERM, which makes the model tend to predict disease cases rather than exclude non-disease ones. This is in accord with our reported lower specificity of AI model (78.69%) compared with manual diagnosis (91.80%), in other words, more people without ERM are labeled as ERM by the AI model.

The choice of threshold of our AI model for positive prediction in this study addressed a balance between sensitivity and specificity. In Fig. 5, the operation points of ophthalmologist 1 and 2 are below or on the ROC curve of AI model, which means the model has a better or equivalent performance. For the diagnosis of ophthalmologist 3, although its operation point is above the curve due to higher specificity, its sensitivity is too low, indicating that its use is biased and limited. Furthermore, in a specific application, the model can be calibrated by choosing a threshold corresponding to a preferred sensitivity while achieving a comparable specificity with ophthalmologist’s diagnosis, and vice versa. Thus our AI model could be used as a possible alternative of acceptable accuracy at a relatively low cost when there is difficult access to ophthalmological specialists or advanced examination. Another limitation of our study is that we have not validated the use of this AI model in populational screening, which could be conducted in our sequential studies.

## Supplementary Information

Supplementary Information.
