# Supplementary material for: Artificial intelligence-based detection of epimacular membrane from color fundus photographs
Source: Sci Rep. 2021 Sep 29;11:19291. doi: 10.1038/s41598-021-98510-x (PMC8481557; doi:10.1038/s41598-021-98510-x)
Supplement: Supplementary file 1 — Supplementary Information. [file 41598_2021_98510_MOESM1_ESM.pdf]

Artificial intelligence-based detection of epimacular membrane from color fundus photographs

Enhua Shao[1,†], Congxin Liu[2,†], Lei Wang[1], Dan Song[1], Libin Guo[1], Xuan Yao[2], Jianhao Xiong[2], Bin Wang[2], and Yuntao Hu[1,*]

[1]Beijing Tsinghua Chang Gung Hospital, Department of Ophthalmology, China
[2]Beijing Eaglevision Technology Co., Ltd, Beijing, China
[*]ythu203@163.com
[†]These authors contributed equally to this work

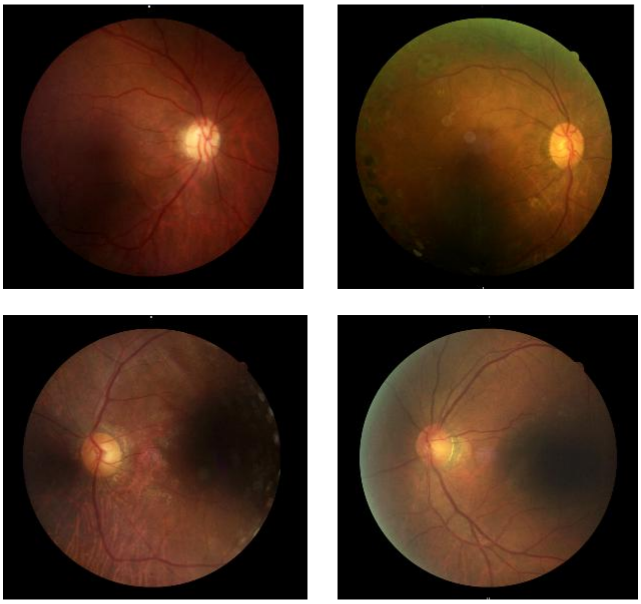

Supplementary Figure 1 Example of fundus images mi-classified by AI model.
